# Evaluation of Droplet Digital PCR Assay for the Diagnosis of Candidemia in Blood Samples

**DOI:** 10.3389/fmicb.2021.700008

**Published:** 2021-09-03

**Authors:** Biao Chen, Yingguang Xie, Ning Zhang, Wenqiang Li, Chen Liu, Dongmei Li, Shaodong Bian, Yufeng Jiang, Zhiya Yang, Renzhe Li, Yahui Feng, Xiaojie Zhang, Dongmei Shi

**Affiliations:** ^1^The Laboratory of Medical Mycology, Jining No. 1 People's Hospital, Jining, China; ^2^Postdoctoral Mobile Station of Shandong University of Traditional Chinese Medicine, Jinan, China; ^3^Intensive Care Unit, Jining No. 1 People’s Hospital, Jining, China; ^4^Department of Microbiology and Immunology, Georgetown University Medical Center, Washington, DC, United States; ^5^Clinical Laboratory, Jining No. 1 People’s Hospital, Jining, China; ^6^Clinical Medicine College, Jining Medical College, Jining, China; ^7^Department of Dermatology, Jining No. 1 People’s Hospital, Jining, China

**Keywords:** droplet digital PCR, diagnosis, candidemia, blood samples, sensitivity, specificity

## Abstract

Numerous studies have shown that droplet digital PCR (ddPCR) is a promising tool for the diagnosis of pathogens, especially in samples with low concentrations of pathogenic DNA. An early diagnosis of candidemia is critical for the effective treatment of patients. In this study, we evaluated the sensitivity and specificity of ddPCR assay for *Candida* DNA detection both *in vitro* by mixing fungal cells with human blood and *in vivo* by analyzing blood samples from infected mice and patients with suspected candidemia. The results showed that ddPCR assay could detect a minimum of 4.5 DNA copies per reaction in blood samples. ddPCR showed higher sensitivity and specificity for *Candida* DNA detection than traditional culture and quantitative PCR (qPCR) methods and also exhibited significantly better positive and negative predictive values than the culture and qPCR methods that were commonly used in clinical practice. Hence, our study demonstrates that ddPCR assay is a promising method for the timely diagnosis of candidemia and could be useful for monitoring the treatment of candidemia.

## Introduction

Candidemia is a leading cause of fungal infections among neonates and infants, and it also affects immunocompromised adults (Golan et al., 2005; Mantadakis et al., 2018). The incidence of candidemia is 13.3 per 100,000 people, and the mortality ranges from 36 to 50% (Ngamchokwathana et al., 2021). Given the rapid and fatal course of candidemia, timely and effective treatment depends on rapid and accurate diagnosis of this invasive fungal infection (Pfaller and Diekema, 2007; Schroeder et al., 2020).

The traditional procedure for diagnosing candidemia relies on blood cultures for the isolation of *Candida* spp. However, this method has low sensitivity and requires large volumes of blood (Clancy and Nguyen, 2013; Pappas et al., 2018). The growth, isolation, and identification of *Candida* spp. routinely take 24–48h. This time-consuming approach takes even longer time when dealing with slower-growing fungal species, which will prevent the timely diagnosis and the initiation of appropriate anti-fungal treatment. The first 12–48h post-infection is a critical period since delaying treatment significantly increases the mortality of candidemia (Tumbarello et al., 2007; Tulasidas et al., 2018). Hence, the broad-spectrum empirical anti-fungal treatment is frequently used for high-risk patients. However, this will unnecessarily increase costs and risk of adverse reactions in these patients (Tumbarello et al., 2007; de Pauw et al., 2008).

Recent efforts in the development of early diagnostic methods have focused on the identification of molecular markers of pathogens. Also, quantitative PCR (qPCR)-based methods for DNA detection have become more popular than other non-culture methods. They facilitate the rapid diagnosis of bloodstream fungal infections, allowing for the initiation of species-oriented therapy as soon as 6h after the onset of sepsis (Mcmullan et al., 2008). However, the sensitivity, accuracy, and replicability of these techniques do not fulfill the requirements of clinical practice, especially in the cases of samples with low abundance of pathogen DNA or with insufficient volumes of blood, as is common in premature or young infants (Nyaruaba et al., 2019).

Droplet digital PCR (ddPCR), based on water–oil emulsion droplet technology, is a new PCR method for nucleic acid detection that allows more accurate quantification of DNA templates (Schell et al., 2012). Each sample is partitioned into approximately 20,000 droplets before being subjected to the procedure. Ideally, each droplet contains one target molecule or none. Unlike qPCR, there is no need to establish a standard curve. The number of droplets with their amplification products is counted at the end of amplification, allowing for an estimate of template concentration based on Poisson’s Law of Small Numbers. With a high sensitivity to detect low copies of DNA, ddPCR assay has been applied in several viral infections such as *human papilloma virus* (Schiavetto et al., 2021), *hepatitis B virus* (Lillsunde), chromosomally integrated *human herpes virus* 6 (Sedlak et al., 2014), and *Mycobacterium* spp. detection for tuberculosis (Yang et al., 2017) and leprosy (Cheng et al., 2019). However, the clinical utility of ddPCR assay for fungal detection remains unclear. To further optimize the ddPCR assay for the detection of *Candida* spp., the diagnostic performance of ddPCR assay was compared with conventional culture and qPCR assays using blood samples from mice with experimental candidemia and patients with suspected candidemia. The specificity of ddPCR for *Candida* spp. was also estimated using non-*Candida* fungal templates *in vitro*.

## Materials and Methods

### Fungal and Bacterial Strains and Culture Conditions

Several typical fungal and bacterial strains, including *Candida albicans* (SC 5314), *C. tropicalis* (CBS 8072), *C. parapsilosis* (ATCC 22019), *C. krusei* (CBS 6451), *Trichophyton rubrum* (ATCC 4438), *Aspergillus fumigatus* (MAY 3626), *Staphylococcus aureus* (ATCC 25923), *Escherichia coli* (ATCC 35218), *Pseudomonas aeruginosa* (ATCC 27853), and *Streptococcus pneumoniae* (ATCC 49619), were obtained from the Laboratory of Medical Mycology, Jining No. 1 People’s Hospital, Shandong, China. Fungal cells were inoculated on Sabouraud-glucose-agar (SDA) plates for 72h at 30°C, and bacterial strains were cultured on blood agar plates (Jinan Baibo Biotechnology Co., Ltd.) for 24h at 37°C.

### DNA Extraction

Fungal DNA extraction was performed according to the manufacturer’s (OMEGA) instructions. Each blood sample (0.25ml) was transferred into sterile micro-centrifuge tubes containing 25μl OB protease solution and 250μl BL buffer. After incubation at 65°C for 10min, 260μl ethanol was added to each blood sample, which was then vortexed at the maximum speed for 20s and transferred into HiBind® DNA mini-columns and centrifuged at 10,000*g* for 1min. In a new collection tube, 500μl HBC buffer was added to a DNA mini-column. After centrifuging again at 1,000*g* for 1min, DNA was washed with 700μl wash buffer and finally collected in a nuclease-free 2-ml micro-centrifuge tube with 200μl elution buffer at 65°C at 13,000*g* for 1min. The DNA samples were immediately stored at −20°C until use. All laboratory equipment was disinfected and decontaminated using UV-treatment prior to DNA preparation. Laboratory equipment and surfaces were also regularly disinfected using 10% bleach and 70% ethanol before conducting any analysis.

### Quantitative PCR

*Candida albicans* in blood samples was detected by a qPCR assay using the primers (forward: 5'-TCAAAACTTTCAACAACGGATCTC-3'; and reverse: 5'-CGCATTTCGCTGCGTTCT-3') synthesized by China Electronics Huada Technology Co. Ltd. In a total volume of 25μl qPCR reaction, 12.5μlTB Green Premix Ex Taq II was mixed with 4.84μl of DNA (66ng), 1μl (25pmol) each of forward primer and reverse primer, and 5.66μl H_2_O. The amplification was performed under the following conditions: the initial denaturation at 95°C for 30s and 40cycles of amplification at 95°C for 5s and 57°C for 30s. Bio-Rad CFX Maestro detection system was used for amplification, detection, and data analysis. Internal, positive, and negative controls were included in each experiment. To avoid the risk of false positive results due to laboratory contamination, all the experimental setups were performed in a biosafety cabinet.

### Droplet Digital PCR

All ddPCR assays were conducted using QX200 Droplet Digital PCR system (Bio-Rad, United States) in a 22-μl volume system. The primers and probe for *C. albicans* were as follows: forward primer 5'-TCAAAACTTTCAACAACGGATCTC-3'; reverse primer 5'-CGCATTTCGCTGCGTTCT-3', and the probe: 5'-TGGTTCTCGCATCGAT-3'. The probe was labeled with FAM. The probe and two primers were synthesized by China Electronics Huada Technology Co. Ltd. Internal, positive, and negative controls were included in each ddPCR run. Each reaction contained 11μl Bio-Rad 2×ddPCR Supermix, 1.1μl forward primer (18μM) and 1.1μl reverse primer (18μM), 1.1μl probe (5μM), 4.84μl template DNA (66ng), and 2.86μl H_2_O. The PCR mix and sample DNA were thoroughly mixed before loading to a droplet generator cartridge. After droplet generation oil was added, the DNA mixtures were placed into a droplet generator (Bio-Rad, United States). Droplet-partitioned samples were transferred to a ddPCR 96-well plate, which was then sealed at 180°C using a PX1TM PCR plate sealer (Bio-Rad) before being amplified in a thermal cycler. The PCR annealing temperature was optimized at 57°C, as this temperature provided a clear separation between DNA-positive and negative droplets in the preliminary analysis. PCR was performed in a T100TM thermal cycler under the following conditions: 95°C for 10min, followed by 40cycles of 94°C for 30s and 57°C for 60s, and the final extension at 98°C for 10min. Moreover, a ramp rate was set up at 2°C/s in the PCR program for every amplification step. After amplification, droplets remained at 4°C for at least 30s. DNA targets in each droplet were quantized by thermal cycling and analyzed by the Bio-Rad QX200TM droplet reader. To limit laboratory contamination, all the setup procedures before thermal cycler use were performed in a biosafety cabinet.

### Estimation of the Limit of Detection and Specificity

To calculate the limit of detection (LOD) of *C. albicans* DNA in blood samples, 10-fold dilutions of 1×10^−1^ng/μl stock of *C. albicans* DNA were prepared with PBS. The final range of DNA dilution was 10^−1^–10^−7^ from the *C. albicans* stock concentration (1ng/μl) in ddPCR analysis. The specificity of ddPCR for *C. albicans* DNA detection was evaluated with the DNA extractions from *C. tropicalis*, *C. parapsilosis*, *C. krusei*, *Staphylococcus aureus*, *Escherichia coli*, *Pseudomonas aeruginosa*, *Streptococcus pneumoniae*, and *Klebsiella pneumoniae* using the same primer set for *C. albicans* assay. DNA stock used in this experiment was quantified using NanoDrop One/OneC (Thermo, USA).

### Experimental Candidemia in Mice With *C. Albicans*

Experimental candidemia was established in mice by intravenous challenge with cultured cells of *C. albicans*. In each experiment, BALB/c female mice (aged 4–8 weeks) were infected by the tail vein injection of *C. albicans* cells in 500μl sterile PBS at 2×10^6^ CFU, 2×10^5^ CFU, or 2×10^4^ CFU, respectively. Each concentration of *C. albicans* was injected into 15 female mice. Animals were randomly assigned to different groups. At subsequent time points (days 1, 3, and 7 post-infection), the mice were euthanized by carbon dioxide. The blood samples were obtained by orbital puncture, collected in EDTA tubes, and divided into three parts for quantitative culturing on SDA agar plates, conventional qPCR and ddPCR testing.

### Blood Sampling From Patients With Suspected Candidemia

Forty-five blood samples of hospitalized patients with suspected candidemia were collected between January 2019 and April 2020 at Jining No. 1 People’s Hospital (Jining, China). The blood samples were collected into EDTA tubes for immediate detection or storage at −80°C for further use. All samples were ultimately used for culture, and the extracted DNA samples were used for qPCR and ddPCR analyses.

### Ethics Statement

The study was approved by Jining Medical College and Jining No. 1 People’s Hospital, Shandong, China (Approval No. 2020–028). The guidelines given by the Genetic Risk Prediction Studies were followed. Written informed consent was obtained from each participant or from legal guardians of any minors. Meanwhile, all the animal experiments were conducted as per the guidelines of the Animal Ethics Committee of Jining No. 1 People’s Hospital. The animal study was reviewed and approved by the Jining Medical College, Shandong.

### Statistical Analysis

We considered the qPCR or culture assay as “gold standard” for fungal identification (Momin et al., 2020). Then, we used these techniques to estimate positive detection rates, sensitivities, specificities, and positive predictive and negative predictive values of ddPCR in suspected candidemia. IBM SPSS Statistics for Windows, Version 24.0 (IBM Corp., Armonk, NY) was used for all statistical analyses.

## Results

### Designed ddPCR Assay Is Specific to *C. Albicans*

**Figure 1:**
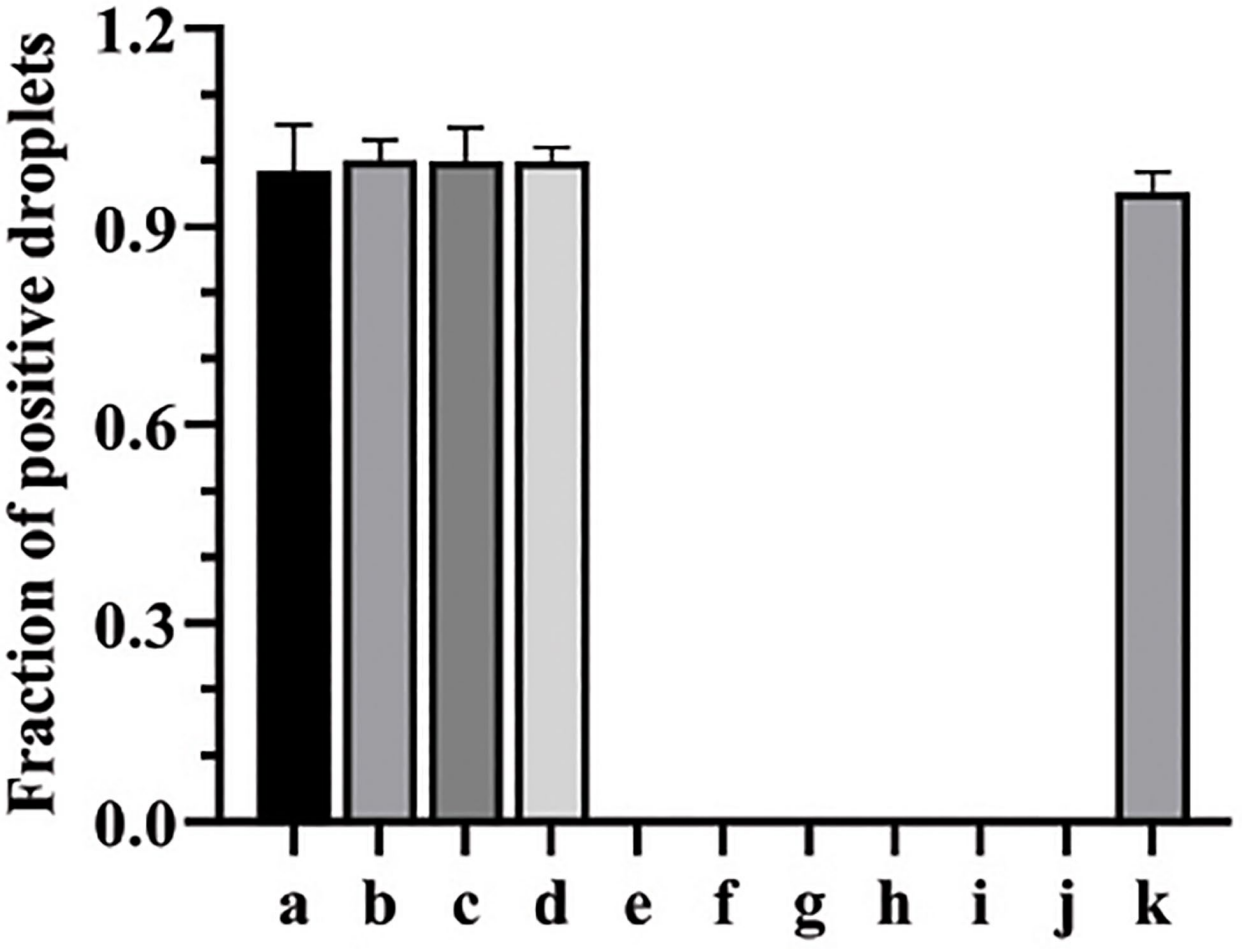
The specificity of ddPCR assay for the detection of *Candida albicans*. a, b, c, d, e, f, g, h, i, and g represent the fraction of positive droplets of *Candida tropicalis*, *C. parapsilosis*, *C. krusei*, *C. albicans*, *Trichophyton rubrum*, *Aspergillus fumigatus*, *Staphylococcus aureus*, *Escherichia coli*, *Pseudomonas aeruginosa*, and *Streptococcus pneumoniae*, respectively. k represents positive control (*C. albicans*).

The primers and probe designed in this study for both qPCR and ddPCR were genus-specific for *C. albicans*. To analyze the general specificity of the ddPCR method, we used the same primers, probe, and amplification procedure against genomic DNA preparations from other fungal and bacterial species. These other pathogens are also commonly found in bacteremia or candidemia blood samples. The ddPCR results showed that *C. tropicalis*, *C. parapsilosis*, and *C. krusei* could be amplified; however, all the bacterial samples were negative with *C. albicans*-specific primers, even though the concentrations of DNA template were 66ng per ddPCR reaction. Meanwhile, the negative controls showed no amplified product. Since all the bacterial samples with non-target DNA were negative, ddPCR assay showed good specificity in detecting *Candida* spp. (Figure 1).

### ddPCR Has Improved Quantitative Range in Comparison to qPCR

**Figure 2:**
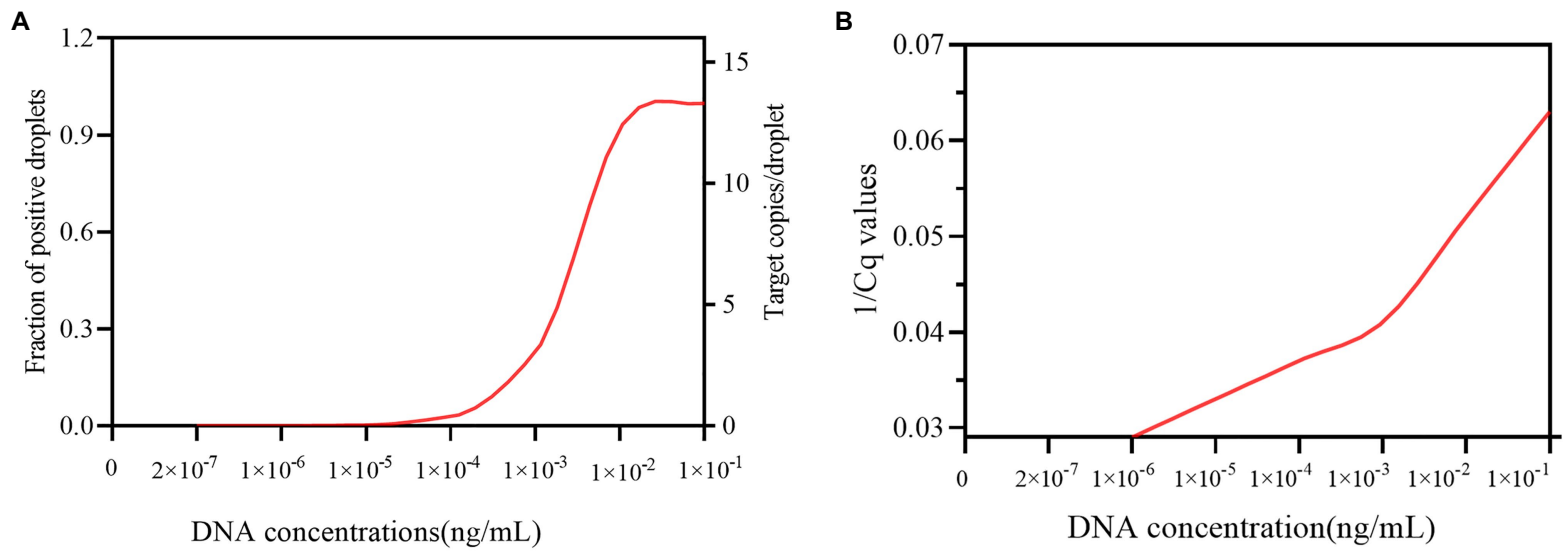
The sensitivity of the ddPCR and qPCR assays in detecting candidemia. **(A)** Shows the sensitivity of ddPCR in detecting candidemia. X-axis represents the theoretical dynamic range of DNA concentration (2×10^−7^–1×10^−5^ng/ml). Y-axis on the left represents the fraction of positive droplets detected by ddPCR. Y-axis on the right represents target copies/droplet detected by ddPCR. **(B)** Shows the sensitivity of qPCR in detecting candidemia. X-axis indicates the DNA concentration, ranging from 2×10^−7^–1×10^−1^ng/ml Y-axis indicates the 1/Cq value detected by qPCR.

The minimum concentration necessary for DNA in the blood samples for ddPCR detection was first determined *in vitro* by 10-fold serial dilution from an initial concentration of 1×10^−1^ ng/μL DNA from *C. albicans* strain (SC 5314). The DNA copies per reaction ranged from 142,600 to 0. The fraction of positive droplets ranged from 0.9977 to 0. The target copies/droplet ranged from 15.8058 to 0. And, the results showed that the LOD for ddPCR detection for *C. albicans* was 4.5 copies per reaction, provided by 2×10^−7^-fold dilution sample. When compared with ddPCR, the minimum number of DNA concentration for qPCR must be greater than the number of DNA concentration in 1×10^−6^-fold dilution sample, since the qPCR method requires a 34.3 Cq value (1/Cq value with 0.029) for *C. albicans* detection at the 1×10^−6^-fold dilution point. This Cq value was almost indistinguishable from the 36.7 Cq value (1/Cq value with 0.027) obtained from the negative control (Figure 2 and Supplementary Table 1). Therefore, the results indicated that the ddPCR method had at least 5-fold higher sensitivity than the qPCR method for *C. albicans* DNA detection in blood samples.

### ddPCR Detected Candidemia *in vivo*

**Figure 3:**
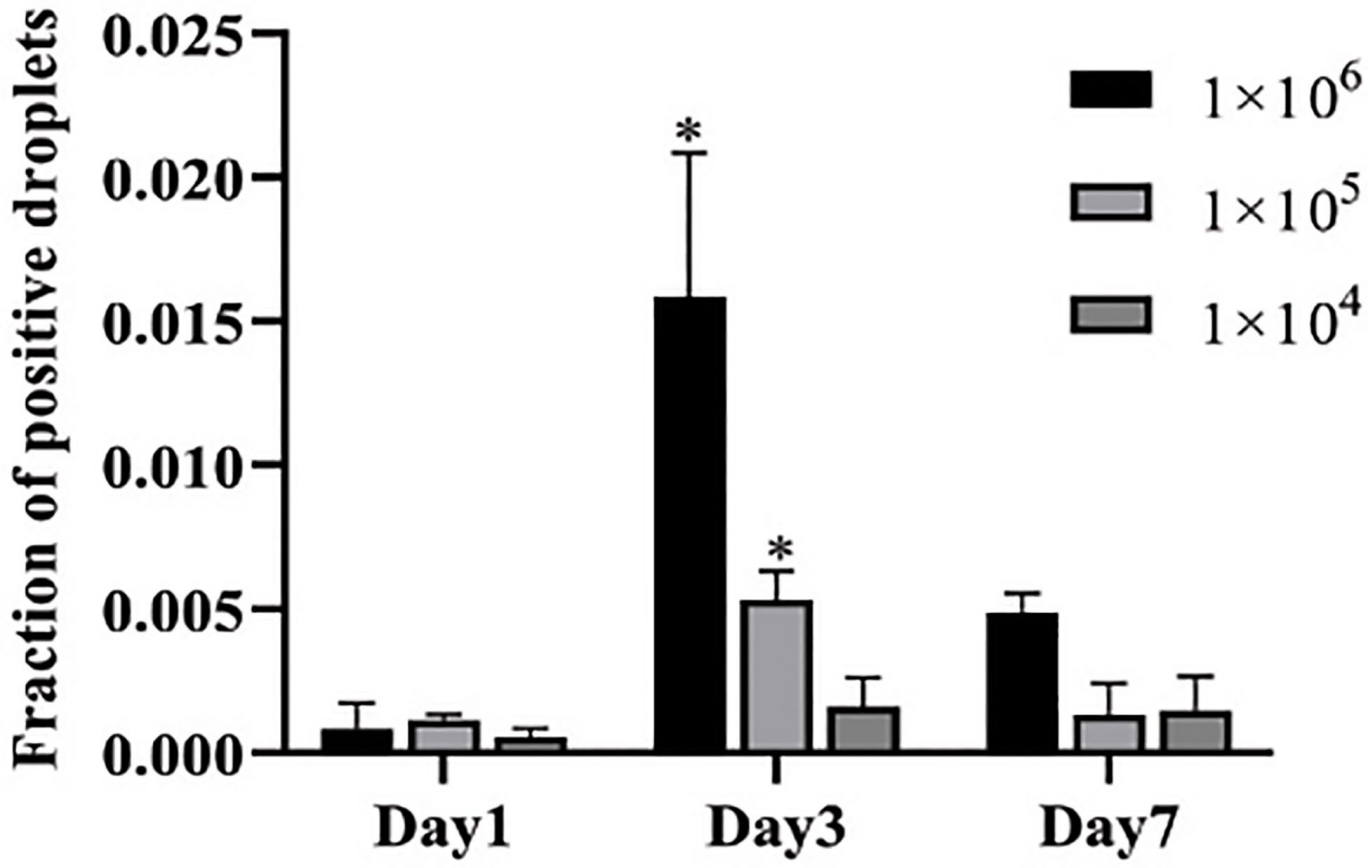
Determination of fraction of positive droplets by ddPCR assay in infected mice. Three concentrations of *Candida* cells were injected into three groups of mice (*n*=5 for each group) *via* tail vein. The copies of *C. albicans* DNA in blood samples are determined at days 1, 3, and 7 post-infection. Y-axis represents fraction of positive droplets. The fraction of positive droplets is significantly higher at day 3 post infection with 1×10^6^ and 1×10^5^ inoculations than at the time points of days 1 and 7. ^*^Present the *p* value <0.05.

To further evaluate the performance of ddPCR in the detection of *C. albicans* infection *in vivo*, we used infected mice to mimic candidemia in humans. Blood samples from infected mice were collected at day 1, 3, and 7 post-infection with different concentrations of *C. albicans* cells. As expected, the detection capacity of fraction of positive droplets by ddPCR method increased with higher dosages of *C. albicans*. For example, at day 1, the fraction of positive droplets were found to be 0.00055, 0.00114, and 0.00085 for inoculations of 1×10^4^CFU, 1×10^5^CFU, and 1×10^6^CFU, respectively (Figure 3). The fractions of positive droplets were 0.00161, 0.00531, and 0.01584 at day 3, and then dropped slightly to 0.00146, 0.00132, and 0.00488 at day 7 post-infection. Indeed, this peak at day 3 was consistent with the peak infection course in the mouse infection model. These mice often died or became seriously ill during the 3–5days post-infection. These results suggested that the ddPCR assay was not only useful for an early diagnosis but also provided prognostic value for candidemia management.

### ddPCR Showed Higher Sensitivity in Detecting *Candida* Among Suspected Candidemia Samples

A total of 45 blood samples from hospitalized patients with suspected candidemia were used to further evaluate the effectiveness of ddPCR in detecting *Candida* in clinical samples. The detection rates of the ddPCR method were also compared with those obtained from culture and qPCR methods. The results showed that the positive detection rates were 44% for culture method (*n*=20), 51% for qPCR (*n*=23), and 73% for ddPCR (*n*=32), respectively, of which 29 patients (64%) were positive either by culture or qPCR method alone, or by both methods, which was inferior to the positive rate of the ddPCR method. When compared with the two other methods, the sensitivity of ddPCR was much higher, with 94 *vs.* 69% for culture and 79% for qPCR method (Table 1). With 91% positive predictive value and 85% negative predictive value, the ddPCR method was more sensitive and efficient than either culture or qPCR method for candidemia diagnosis.

**Table 1:** Positive and negative predictive values, sensitivity and specificity of different diagnostic assays in patients with suspected candidemia (*n*=45, including two neonates).

Diagnostic method	Detection rate (%)	Sensitivity (%)	Specificity (%)	Positive predictive value (%)	Negative predictive value (%)
Culture	44	69	100	100	64
RT-PCR	64	89	76	86	81
ddPCR	73	94	79	91	85

*RT-PCR, Real Time-PCR; and ddPCR, droplet digital PCR*.

## Discussion

This study demonstrated that ddPCR assay facilitated accurate quantification of *Candida* DNA in human blood. As no standard curve is needed, ddPCR also allows for the direct comparison of *Candida* concentrations measured by different laboratories. Previous studies found that variation in quantification between technical replicates was considerably lower for ddPCR than for qPCR (Sedlak et al., 2014; Wu et al., 2018). Hence, ddPCR has advantages over other qPCR methods for pathogen DNA detection in clinical samples. Despite the popularity of qPCR, differences between testing instruments, different agents or suppliers, or variations in the standard curves can confound the interpretation and the portability of results (Chindamporn et al., 2018; Cutarelli et al., 2021).

The ddPCR method showed good reproducibility and high specificity in this study. As expected, the sensitivity of ddPCR assay for *Candida* spp. detection in blood samples was significantly higher than that of culture or qPCR methods. The sensitivity of ddPCR (94%) was higher than the 86% observed in neonates in a previous study (Li et al., 2019). Given that our patients were mainly adults, this difference could be due to quantitative differences of fungal cells in the blood between adults and neonates after the onset of disease (sampling time). The ddPCR method also has other diagnostic advantages over conventional qPCR, including lower sample volume requirements and faster execution times, because the thermocycling times are shorter (De falco et al., 2021).

The specificity of the probe sets used in ddPCR assays for specific microbes is often established using closely related species and unrelated species such as human DNA. The probe in the present study was 100% specific for *Candida* genus and did not hybridize with human DNA. The probe was designed based on two observations. First, about 80% of current systemic fungal infections are caused by *Candida* species; second, approximately 60% of fungal isolates are *C. albicans* (Kim et al., 2020). However, non-*albicans* species, such as *C. tropicalis*, *C. parapsilosis*, *C. glabrata*, and *C. krusei*, are also prevalent (Krcmery and Barnes, 2002; Wisplinghoff et al., 2014). The probe and primer set used in this study could detect DNA from non-*albicans* species, such as *C. tropicalis*, *C. parapsilosis* and *C. glabrata*, which would reduce the likelihood of overlooking the presence of non-*albicans* species in clinical practice. Meanwhile, this probe and primer set based on the *Candida* genus could not detect other fungal pathogens, such as *Aspergillus* spp. and *T. rubrum*, and bacteria such as *S. aureus*, *E. coli*, *P. aeruginosa*, and *S. pneumoniae*, which are commonly found in patients with bacteremia.

Both high sensitivity and specificity in ddPCR could facilitate the studies on other fungal detections in future. For example, an assay to identify fungal species in a single run of ddPCR assay could be developed using two probes, of which one probe could be based on the genus and the second probe could be based on the particular species. We will continue to evaluate the sensitivity and specificity of ddPCR in larger candidemia cohorts, and in other types of clinical samples (*e.g.*, urine or BAL). Given its higher sensitivity, a prospective clinical study using the ddPCR assay for the identification of patients at high risk for invasive fungal infections is underway.

The high specificity and sensitivity of ddPCR facilitates its application in the early diagnosis of candidemia. In this study, two blood samples were obtained from infants suspected with candidemia. Both infants were detected positive by ddPCR assay but negative by culture and qPCR assays. Following the diagnosis of candidemia based on the ddPCR assay, these two infant patients were immediately given anti-fungal treatment and quickly cured. In order to avoid false negative result, a series of diluted samples need to be prepared and detected by ddPCR assay in the early diagnosis of candidemia.

## Data Availability Statement

The raw data supporting the conclusions of this article will be made available by the authors, without undue reservation.

## Ethics Statement

The studies involving human participants were reviewed and approved by the Jining Medical College and Jining No. 1 People’s Hospital, Shandong, China (2020–028). Written informed consent to participate in this study was provided by the participants’ legal guardian/next of kin. The animal study was reviewed and approved by the Jining Medical College, Shandong, China (2020–028).

## Author Contributions

BC was mainly for investigation, formal analysis, and writing-original draft. NZ was mainly for investigation and formal analysis. WL, DL, CL, SB, YJ, ZY, RL, and YF were mainly for collecting samples. YX, XZ, and DS were mainly for designing the study, funding acquisition, investigation, and review and editing. All authors contributed to the article and approved the submitted version.

## Conflict of Interest

The authors declare that the research was conducted in the absence of any commercial or financial relationships that could be construed as a potential conflict of interest.

## Publisher’s Note

All claims expressed in this article are solely those of the authors and do not necessarily represent those of their affiliated organizations, or those of the publisher, the editors and the reviewers. Any product that may be evaluated in this article, or claim that may be made by its manufacturer, is not guaranteed or endorsed by the publisher.
