# Supplementary material for: Evaluation of Droplet Digital PCR Assay for the Diagnosis of Candidemia in Blood Samples
Source: Front Microbiol. 2021 Sep 3;12:700008. doi: 10.3389/fmicb.2021.700008 (PMC8480469; doi:10.3389/fmicb.2021.700008)
Supplement: Supplementary file 1 [file Table_1.docx]

**Supplementary Table 1.** Sensitivity of the ddPCR and qPCR assays in detecting candidemia

| Sample no. | Concentration of transcripts (ng/mL) | ddPCR | | | | qPCR | | |
| --- | --- | --- | --- | --- | --- | --- | --- | --- |
|  |  | Copies/20μL（Mean ± SD） | Fraction of positive droplets | Target copies/droplet | +/- | Cq （Mean ± SD） | 1/Cq | +/- |
| 1 | 1×10^-1^ | 142466.7±416.3 | 0.9977 | 15.80580 | + | 15.8±0.1 | 0.063 | + |
| 2 | 1×10^-2^ | 72933.3±305.5 | 0.9542 | 7.85544 | + | 19.2±0.3 | 0.052 | + |
| 3 | 1×10^-3^ | 6020.0±52.9 | 0.2277 | 0.62966 | + | 24.1±0.1 | 0.041 | + |
| 4 | 1×10^-4^ | 680.0±26.5 | 0.0289 | 0.07379 | + | 27.1±0.2 | 0.037 | + |
| 5 | 1×10^-5^ | 62.7±7.6 | 0.0028 | 0.00666 | + | 30.3±0.1 | 0.033 | + |
| 6 | 1×10^-6^ | 12.6±1 | 0.0005 | 0.0010 | + | 34.3±0.2 | 0.029 | + |
| 7 | 2×10^-7^ | 4.5±0.8 | 0.0002 | 0.0005 | + | 37.0±0.2 | 0.027 | - |
| 8 | 1×10^-7^ | 0.0±0.0 | 0 | 0 | - | 36.2±0.2 | 0.027 | - |
| 9 | NTC | 0.0±0.0 | 0 | 0 | - | 36.7±0.2 | 0.027 | - |

Abbreviations: Cq, quantification cycle; SD, standard deviation.

+indicates positive; -indicates negative.
